# *MDM4* SNP34091 (rs4245739) and its effect on breast‐, colon‐, lung‐, and prostate cancer risk

**DOI:** 10.1002/cam4.555

**Published:** 2015-10-16

**Authors:** Liv B. Gansmo, Pål Romundstad, Einar Birkeland, Kristian Hveem, Lars Vatten, Stian Knappskog, Per Eystein Lønning

**Affiliations:** ^1^ Section of OncologyDepartment of Clinical ScienceUniversity of BergenBergenNorway; ^2^ Department of OncologyHaukeland University HospitalBergenNorway; ^3^ Faculty of MedicineDepartment of Public HealthNorwegian University of Science and TechnologyTrondheimNorway

**Keywords:** Cancer risk, MDM4, population based, SNP309, SNP34091

## Abstract

The MDM4 protein plays an important part in the negative regulation of the tumor suppressor p53 through its interaction with MDM2. In line with this, *MDM4* amplification has been observed in several tumor forms. A polymorphism (rs4245739 A>C; SNP34091) in the *MDM4* 3′ untranslated region has been reported to create a target site for hsa‐miR‐191, resulting in decreased *MDM4* mRNA levels. In this population‐based case–control study, we examined the potential association between *MDM4* SNP34091, alone and in combination with the *MDM2* SNP309T>G (rs2279744), and the risk of breast‐, colon‐, lung‐, and prostate cancer in Norway. SNP34091 was genotyped in 7,079 cancer patients as well as in 3,747 gender‐ and age‐matched healthy controls. *MDM4* SNP34091C was not associated with risk for any of the tumor forms examined, except for a marginally significant association with reduced risk for breast cancer in a recessive model (OR = 0.77: 95% CI = 0.59–0.99). Stratifying according to *MDM2* SNP309 status, we observed a reduced risk for breast cancer related to *MDM4* SNP34091CC among individuals harboring the *MDM2* SNP309GG genotype (OR = 0.41; 95% CI = 0.21–0.82). We conclude, *MDM4* SNP34091 status to be associated with reduced risk of breast cancer, in particular in individuals carrying the *MDM2* SNP309GG genotype, but not to be associated with either lung‐, colon‐ or prostate cancer.

## Introduction

The tumor suppressor p53 plays a pivotal role in many physiological processes, including metabolism and maintenance of genomic stability. In order to allow normal cell proliferation and to maintain cell viability during absence of stress signals, the activity of p53 is kept under strict control, predominantly by the protein product of the murine double minute 2 gene, *MDM2*, and its homolog *MDM4*, acting in concert 1. It is well established that MDM2 and p53 are linked in an autoregulatory negative feedback loop, where p53 transcriptionally induces *MDM2* and MDM2 downregulates p53 2, mainly by direct inhibition and/or proteolytic degradation 3, 4, 5. Although MDM4 alone is unable to target p53 for ubiquitin‐proteasome‐dependent degradation 6, the MDM2/MDM4 heterodimer has been shown more potent degrading the p53 protein as compared to the MDM2 homodimer 7, 8. Additionally, using Mdm2/Mdm4/p53 triple knockout MEFs, Yuan and colleagues showed that an Mdm2/Mdm4 heterodimer is required for the E3 ligase activity of Mdm2 9. These data suggest that elevated levels of MDM4 may contribute to reduced p53 activity and tumor development. In line with this, the *MDM4* gene has been found amplified in malignant gliomas with no *TP53* mutations or *MDM2* amplifications 10, 11 as well as in breast cancer 12, and acute lymphoblastic leukemia 13. Furthermore, studies in transgenic mice show that overexpression of Mdm4 induced spontaneous tumor formation and accelerated tumorigenesis 14.

Single‐nucleotide polymorphisms (SNP) affecting the levels of both MDM2 and MDM4 have been reported 15, 16, 17, 18. While *MDM2* SNP309T>G (rs2279744) and SNP285G>C (rs117039649) both affect *MDM2* transcription, *MDM4* SNP34091A>C (rs4245739) has been found to affect *MDM4* mRNA stability and protein levels 17, 18. SNP34091 is located in the 3′ untranslated region of *MDM4*, and was found to create a functional target site for hsa‐miR‐191 and hsa‐miR‐887. Both miRs bind to the *MDM4* SNP34091 C‐allele with higher affinity than to the *MDM4* SNP34091 A‐allele, leading to miR‐mediated decrease in MDM4 protein levels in cells carrying the *MDM4* SNP34091C variant 17, 18. Genotype AA was recorded to be more frequent in patients with high‐grade than low‐grade ovarian carcinoma 18. Furthermore, previous studies have indicated the SNP34091C allele to be associated with a reduced risk for non‐Hodgkin lymphoma 19, breast cancer 20, esophageal squamous cell carcinoma 21, and prostate cancer 22.

Contrasting these results, genome wide association studies (GWAS) reported the C allele to be associated with an *increased* risk for estrogen receptor negative, and in particular, triple negative breast cancer 23, 24, 25.

In this study, we assessed the impact of *MDM4* SNP34091 status on the risk of cancer of the breast, lung, prostate, and colon in a large population‐based cohort of Caucasian descent.

## Materials and Methods

### Study population

From the population‐based Cohort of Norway (CONOR) study 26, we genotyped 7079 incident cancer cases and 3747 healthy controls, described in detail previously 27. Thus, we examined the potential effect of *MDM4* SNP34091A>C by analyzing the four major cancer forms; breast (*n* = 1,717), lung (*n* = 1,331), colon (*n* = 1,531), and prostate (*n* = 2,500). On the basis of previously published allele frequencies in healthy controls and breast cancer cases 23, we found our study design to provide adequate statistical power (β‐values ranging from 0.83 to 0.95 for the four cancer sample sets, given an α‐value of 0.05).

### *MDM4* SNP34091 genotyping

All samples were genotyped for *MDM4* SNP34091 status using a custom LightSNiP assay (TIB MOLBIOL Syntheselabor GmbH, Berlin, Germany) on a LightCycler 480 II instrument (Roche, Basel, Switzerland). The reactions were performed in a final reaction volume of 10 μL, containing 1 μL LightCycler^®^FastStart DNA Master HybProbe mix (Roche Diagnostics), 0.5 μL LightSNiP mix (TIB MOLBIOL), 3 mmol/L MgCl_2_ and 10–50 ng DNA. The thermocycling and melting curve conditions were as follows: 10 min initial denaturation/activation at 95°C, followed by 45 cycles of denaturation at 95°C for 10 sec, annealing for 10 sec at 60°C and elongation at 72°C for 15 sec. Subsequent to the thermocycling amplification the high‐resolution melting (HRM) step was initiated with a denaturation step at 95°C for 30 sec, followed by melting from 40°C to 75°C with a ramp rate of 0.19°C/sec and finally a cooling step at 40°C for 30 sec. The HRM curve profiles were analyzed by the Melt Curve Genotyping software (version 1.5) on the LightCycler^®^ 480 II instrument (Roche Diagnostics).

### Statistical analysis

Potential deviations from Hardy–Weinberg equilibrium were assessed by calculating the expected genotype distribution based on the observed allele frequencies and comparing the output with the observed genotype distribution using Chi‐square tests.

Potential associations between *MDM4* SNP34091 and the risk of any of the cancer types tested as well as cancer risk within different subgroups were estimated by calculating Odds Ratios (OR) with 95% confidence intervals (CI) and logistic regression adjusting for sex and age. In addition, for colon‐ and lung cancer, overall calculations were performed including both genders using the Mantel–Haenszel test (sex adjusted).

All statistical analyses were performed using the IBM SPSS 22 software package (IBM Corp, Armonk, NY, USA) and Stata 13.0 for Windows (Stata Corp, College Station, TX, USA). All *P*‐values are given as two‐sided.

## Results

### Distribution of *MDM4* SNP34091

In this study, 7,079 cancer cases and 3,747 healthy controls were analyzed for *MDM4* SNP34091 status. Among the healthy individuals, the percentages harboring the three different genotypes (*MDM4* SNP34091AA, AC, and CC) were recorded to be 54.5%, 38.4%, and 7.1%, respectively. The genotype frequencies were found to be in Hardy–Weinberg equilibrium (*P *>* *0.9). A comprehensive overview of the *MDM4* SNP34091 distribution in the healthy controls as well as the four cancer types analyzed is given in Table 1. Among the healthy controls, no substantial gender difference with respect to genotype distribution was observed (*P *=* *0.193).

**Table 1:** *MDM4* SNP34091 distribution and cancer risk

Cases/controls	Genotype			OR (95% CI)	*P*‐value	OR (95% CI)	*P*‐value
	SNP34091 *n* (%)			SNP34091		SNP34091	
	AA	AC	CC	CC versus AA+AC		CC+AC versus AA	
Controls	2042 (54.5)	1439 (38.4)	266 (7.1)	1.00	–	1.00	–
Women	1021 (54.6)	703 (37.6)	146 (7.8)	1.00	–	1.00	–
Men	1021 (54.4)	736 (39.2)	120 (6.4)	1.00	–	1.00	–
Colon cancer^a^	823 (53.8)	600 (39.2)	108 (7.1)	1.04 (0.82–1.32)	0.737	1.04 (0.93–1.18)	0.484
Women^b^	429 (55.1)	293 (37.7)	56 (7.2)	1.02 (0.73–1.41)	0.919	1.01 (0.85–1.20)	0.941
Men^c^	394 (52.3)	307 (40.8)	52 (6.9)	1.09 (0.78–1.54)	0.601	1.10 (0.92–1.30)	0.295
Lung cancer^a^	715 (53.7)	515 (38.7)	101 (7.6)	1.11 (0.87–1.41)	0.396	1.03 (0.91–1.17)	0.662
Women^b^	264 (53.1)	194 (39.0)	39 (7.9)	1.05 (0.73–1.53)	0.781	1.07 (0.87–1.30)	0.535
Men^c^	451 (54.1)	321 (38.5)	62 (7.4)	1.19 (0.86–1.64)	0.288	1.01 (0.86–1.19)	0.912
Prostate cancer^c^	1412 (56.5)	927 (37.1)	161 (6.4)	1.01 (0.79–1.29)	0.946	0.92 (0.82–1.04)	0.182
Breast cancer^b^	966 (56.3)	643 (37.5)	108 (6.3)	0.77 (0.59–0.99)	0.045	0.93 (0.82–1.07)	0.317

a Sex and age adjusted (logistic regression).

b Calculations with female controls only, age adjusted.

c Calculations with male controls only, age adjusted.

### *MDM4* SNP34091 status and cancer risk in four major cancer forms

**Figure 1:**
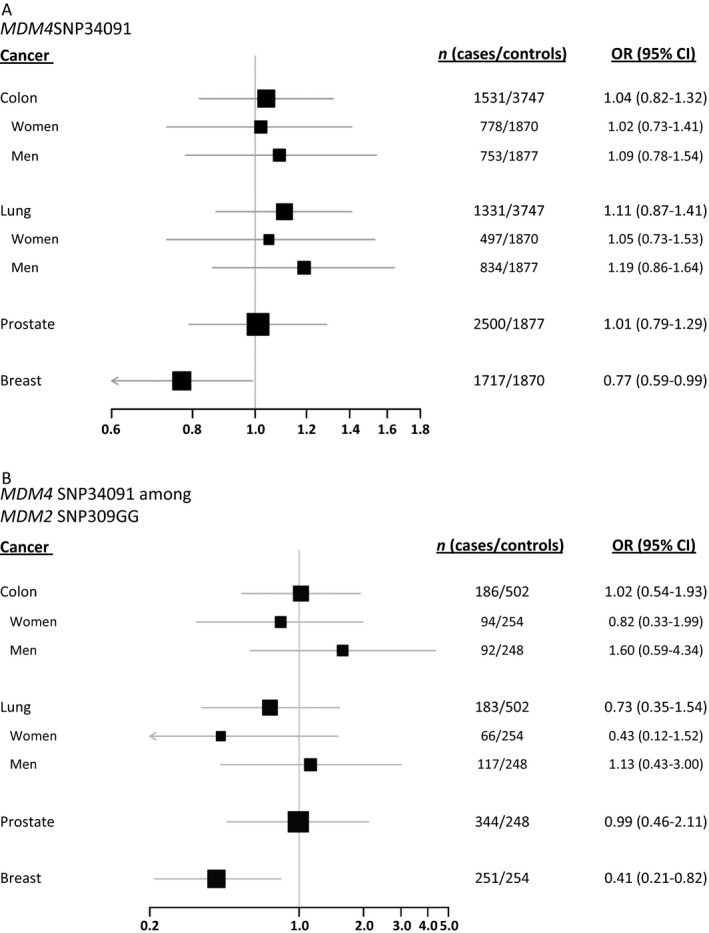
Impact of *MDM4* SNP34091 on cancer risk. Forest plots showing the effect of SNP34091 on cancer of the colon, lung, prostate, and breast, as compared to healthy controls, among the total study population (A) and among individuals harboring the *MDM2* SNP309GG genotype (B).

In order to assess the potential impact of *MDM4* SNP34091 status on cancer risk, we compared the frequency of the *MDM4* SNP34091 genotypes among breast‐ (*n* = 1,717), lung‐ (*n* = 1,331), colon‐ (*n* = 1,531), and prostate cancer (*n* = 2,500) patients to healthy controls (*n* = 3,747). We observed no significant correlation between *MDM4* SNP34091 status and the risk of either cancer in the colon, lung, or prostate, either in a dominant or a recessive model (SNP34091 CC+AC vs. AA, or CC vs. AA+AC, respectively). Furthermore, analyzing tumors of the right or the left side of the colon separately, revealed no significant effect of *MDM4* SNP34091 status and cancer risk in either of the two groups (Table S1). We observed, however, a marginally significant association with reduced risk for breast cancer among individuals harboring the SNP34091CC genotype (recessive model; OR = 0.77; 95% CI = 0.59–0.99; Table 1, Fig. 1A).

Since 92.6% of the lung cancer patients (from whom we had data) were smokers, excluding nonsmokers from the analysis had no impact on the estimates (Table S2).

### Potential interactions between *MDM4* SNP34091 status and *MDM2* promoter SNPs

Previously, we assessed SNP status of the MDM4 partner MDM2 across the same population of cancer patients and healthy controls 27. While the *MDM2* SNP309GG genotype has been associated with a nonsignificantly increased risk for breast cancer, breast cancer patients carrying the SNP309GG genotype have been found particularly sensitive to cancer risk reduction by a second *MDM2* SNP (SNP285G>C) 27. This observation has also been recorded in another separate sample set of breast cancer patients 16.

Since MDM4 forms a heterodimer with MDM2 and promotes MDM2‐mediated polyubiquitination and subsequent degradation of p53, we investigated potential interactions/synergistic effects between *MDM4* SNP34091 and *MDM2* SNPs with respect to cancer risk. Stratifying according to *MDM2* SNP309 status (SNP309TT, SNP309TG, and SNP309GG) we found the *MDM4* SNP34091CC genotype (recessive model) to be significantly associated with reduced risk of breast cancer among patients carrying the SNP309GG genotype (OR = 0.41; 95% CI = 0.21–0.82; Table 2, Fig. 1B). Notably, when refining the OR estimates by removing individuals harboring the less frequent *MDM2* SNP285C allele, which antagonizes SNP309G‐induced transcriptional enhancement 16, this negative association became slightly stronger (gender adjusted OR = 0.40; 95% CI = 0.19–0.85; Table S3).

**Table 2:** *MDM4* SNP34091 among *MDM2* SNP309GG

Cases/controls	Genotype			OR (95% CI)	*P*‐value	OR (95% CI)	*P*‐value
	SNP34091 *n* (%)			SNP34091		SNP34091	
	AA	AC	CC	CC versus AA+AC		CC+AC versus AA	
Controls	294 (58.6)	167 (33.3)	41 (8.2)	1.00	–	1.00	–
Women	149 (58.7)	77 (30.3)	28 (11.0)	1.00	–	1.00	–
Men	145 (58.5)	90 (36.3)	13 (5.2)	1.00	–	1.00	–
Colon cancer^a^	111 (59.7)	60 (32.3)	15 (8.1)	1.02 (0.54–1.93)	0.947	0.98 (0.69–1.39)	0.903
Women^b^	56 (59.6)	30 (31.9)	8 (8.5)	0.82 (0.33–1.99)	0.653	1.09 (0.65–1.83)	0.745
Men^c^	55 (59.8)	30 (32.6)	7 (7.6)	1.60 (0.59–4.34)	0.356	1.03 (0.63–1.70)	0.898
Lung cancer^a^	100 (54.6)	73 (39.9)	10 (5.5)	0.73 (0.35–1.54)	0.413	1.18 (0.83–1.68)	0.357
Women^b^	38 (57.6)	25 (37.9)	3 (4.6)	0.43 (0.12–1.52)	0.190	1.17 (0.66–2.10)	0.588
Men^c^	62 (53.0)	48 (41.0)	7 (6.0)	1.13 (0.43–3.00)	0.799	1.30 (0.82–2.05)	0.264
Prostate cancer^c^	184 (53.5)	143 (41.6)	17 (4.9)	0.99 (0.46–2.11)	0.974	1.26 (0.90–1.77)	0.176
Breast cancer^b^	160 (63.8)	77 (30.7)	14 (5.6)	0.41 (0.21–0.82)	0.012	0.75 (0.52–1.10)	0.139

a Sex and age adjusted (logistic regression).

b Calculations with female controls only, age adjusted.

c Calculations with male controls only, age adjusted.

In addition to assessing the effect of *MDM4* SNP34091 within subgroups of *MDM2* SNP309 genotypes, we also explored differences between all possible combinations of *MDM4* SNP34091/*MDM2* SNP309 genotypes. By doing so, we confirmed the *MDM4* SNP34091CC/*MDM2* SNP309 GG genotype to associated with reduced risk of breast cancer (OR = 0.47; 95% CI = 0.24–0.92, when compared with the highest risk genotype (*MDM4* SNP34091AA/*MDM2* SNP309GG), data not shown).

No effect on the risk of any of the other cancer forms with respect to *MDM4* SNP34091 status within the different *MDM2* genotypes, either when stratifying cancer of the colon according to tumors of the right or left side, or when excluding the nonsmokers in lung cancer, was recorded.

## Discussion

In this study, we observed no association between *MDM4* SNP34091 status and the risk for colon‐, prostate‐, or lung cancer, while a marginally significant association with reduced risk of breast cancer was observed. Our observation in breast cancer is similar to, but weaker than the observations of Liu and colleagues, who found the SNP34091 AC and CC genotypes to be significantly associated with reduced breast cancer risk compared with the AA genotype in two different Chinese populations 20. In contrast, GWAS have found an elevated OR for ER negative breast cancer related to the SNP34091C allele in Caucasians but not among Asians 23, 24, 25. Regrettably, information on receptor status was not available for the breast cancer patients examined in this study; thus, a potential effect of SNP43091 status in the minor group of patients harboring ER negative tumors may have been overlooked. Regarding prostate cancer risk, we observed a weak, non‐significant association between *MDM4* SNP34091C and reduced risk in the dominant model. This is in line with data from the majority of individual datasets from European populations, included in a recent large GWAS 22, and mirrors our previous findings related to MDM2 polymorphisms [27, 28, 29].

This study is, to our knowledge, the first population‐based case–control study assessing the impact of *MDM4* SNP34091 on cancer risk in lung‐ and colon cancer. Previous case–control studies assessing this variant in other cancer forms (esophageal squamous cell carcinoma and non‐Hodgkin lymphoma), including breast cancer, have found the SNP34091C allele to be associated with reduced risk, but have all been performed in Chinese populations 19, 20, 21.

Regarding the variations in the results between studies of different ethnic groups, notably, there is a large difference in the distribution of *MDM4* SNP34091 between Europeans and Asians with a MAF of 0.26 and 0.05, respectively [30], possibly affecting the power of studies in Asian populations even though the numbers of patients included are large. Also, a possible explanation for the discrepancy may be yet unknown functional SNP(s) that are in linkage disequilibrium (LD) with SNP34091: There are examples of functional SNPs in LD where the SNPs have different geographical distributions and thus confer diverging risk estimates between Europeans and Asians, for example, the two *MDM2* SNPs; SNP309 and SNP285 [30, 31, 32, 33]. On the other hand, the possibility of publication bias, where case–control studies reporting positive results are favored cannot be excluded.

After stratifying according to *MDM2* SNP309 status, we found a reduced risk for breast cancer among individuals harboring the *MDM4* SNP34091CC/*MDM2* SNP309GG genotype, and this association was stronger after removing individuals harboring the *MDM2* SNP285C allele, previously shown to antagonize SNP309G‐induced transcription elevation 16. Interestingly, the *MDM4* SNP34091C allele, similar to SNP285G>C seems to execute their effects on breast cancer risk among individuals carrying the SNP309GG genotype only 16, 27.

In conclusion, we found no association between the *MDM4* SNP34091 status and risk for lung‐, prostate‐, or colon cancer, and a weak association with breast cancer, applying the candidate gene approach. The latter finding was substantiated by the observation of a seemingly synergistic effect between the *MDM2* SNP309GG and *MDM4* SNP34091AA genotypes on increased risk for breast cancer.

## Conflict of Interest

None declared.

## Supporting information


**Table S1.** *MDM4* SNP34091 distribution and left versus right colon cancer risk.
**Table S2.** *MDM4* SNP34091 distribution and lung cancer risk in smokers.
**Table S3.** *MDM4* SNP34091 among *MDM2* SNP309GG without *MDM2* SNP285C.
