# Supplementary material for: MDM4 SNP34091 (rs4245739) and its effect on breast‐, colon‐, lung‐, and prostate cancer risk
Source: Cancer Med. 2015 Oct 16;4(12):1901–7. doi: 10.1002/cam4.555 (PMC5123711; doi:10.1002/cam4.555)
Supplement: Supplementary file 1 — Table S1. MDM4 SNP34091 distribution and left versus right colon cancer risk. Table S2. MDM4 SNP34091 distribution and lung cancer risk in smokers. Table S3. MDM4 SNP34091 among MDM2 SNP309GG without MDM2 SNP285C. [file CAM4-4-1901-s001.docx]

| **Supplementary Table 1. *MDM4* SNP34091 distribution and left versus right colon cancer risk** | | | | | | | | | | | |
| --- | --- | --- | --- | --- | --- | --- | --- | --- | --- | --- | --- |
| **Cases/** | **Genotype** | | |  | **OR (95% CI)** |  | **p-value** |  | **OR (95% CI)** |  | **p-value** |
| **controls** | **SNP34091 n (%)** | | |  | **SNP34091** |  |  |  | **SNP34091** |  |  |
|  | **AA** | **AC** | **CC** |  | **CC vs. AA+AC** |  |  |  | **CC+AC vs. AA** |  |  |
| **Controls** | 2042 (54.5) | 1439 (38.4) | 266 (7.1) |  | 1.00 |  | - |  | 1.00 |  | - |
| **Women** | 1021 (54.6) | 703 (37.6) | 146 (7.8) |  | 1.00 |  | - |  | 1.00 |  | - |
| **Men** | 1021 (54.4) | 736 (39.2) | 120 (6.4) |  | 1.00 |  | - |  | 1.00 |  | - |
|  |  |  |  |  |  |  |  |  |  |  |  |
| **CRC Left** | 340 (54.0) | 247 (39.2) | 43 (6.8) |  | 0.96 (0.69-1.34) |  | 0.867 |  | 1.02 (0.86-1.21) |  | 0.829 |
| **Women** | 162 (54.7) | 109 (36.8) | 25 (8.5) |  | 1.09 (0.70-1.70) |  | 0.728 |  | 1.00 (0.78-1.27) |  | 1.000 |
| **Men** | 178 (53.3) | 138 (41.3) | 18 (5.4) |  | 0.83 (0.50-1.39) |  | 0.541 |  | 1.05 (0.83-1.32) |  | 0.721 |
|  |  |  |  |  |  |  |  |  |  |  |  |
| **CRC Right** | 446 (53.9) | 325 (39.3) | 57 (6.9) |  | 0.97 (0.72-1.30) |  | 0.881 |  | 1.03 (0.88-1.19) |  | 0.758 |
| **Women** | 244 (56.5) | 163 (37.7) | 25 (5.8) |  | 0.73 (0.47-1.12) |  | 0.156 |  | 0.93 (0.75-1.14) |  | 0.486 |
| **Men** | 202 (51.0) | 162 (40.9) | 32 (8.1) |  | 1.29 (0.86-1.93) |  | 0.224 |  | 1.15 (0.92-1.42) |  | 0.223 |

| **Supplementary Table 2. *MDM4* SNP34091 distribution and lung cancer risk in smokers** | | | | | | | | | | | |
| --- | --- | --- | --- | --- | --- | --- | --- | --- | --- | --- | --- |
| **Cases/** | **Genotype** | | |  | **OR (95% CI)** |  | **p-value** |  | **OR (95% CI)** |  | **p-value** |
| **controls** | **SNP34091 n (%)** | | |  | **SNP34091** |  |  |  | **SNP34091** |  |  |
|  | **AA** | **AC** | **CC** |  | **CC vs. AA+AC** |  |  |  | **CC+AC vs. AA** |  |  |
| **Controls** | 2042 (54.5) | 1439 (38.4) | 266 (7.1) |  | 1.00 |  | - |  | 1.00 |  | - |
| **Women** | 1021 (54.6) | 703 (37.6) | 146 (7.8) |  | 1.00 |  | - |  | 1.00 |  | - |
| **Men** | 1021 (54.4) | 736 (39.2) | 120 (6.4) |  | 1.00 |  | - |  | 1.00 |  | - |
|  |  |  |  |  |  |  |  |  |  |  |  |
| **Lung cancer** | 639 (52.7) | 482 (39.8) | 91 (7.50) |  | 1.06 (0.83-1.36) |  | 0.655 |  | 1.07 (0.94-1.22) |  | 0.289 |
| **Women** | 219 (51.9) | 173 (41.0) | 30 (7.1) |  | 0.90 (0.60-1.36) |  | 0.686 |  | 1.11 (0.90-1.38) |  | 0.330 |
| **Men** | 420 (53.4) | 305 (38.8) | 61 (7.8) |  | 1.23 (0.89-1.70) |  | 0.206 |  | 1.04 (0.88-1.23) |  | 0.670 |

| **Supplementary Table 3. *MDM4* SNP34091 among *MDM2* SNP309GG without *MDM2* SNP285C** | | | | | | | | | | | |
| --- | --- | --- | --- | --- | --- | --- | --- | --- | --- | --- | --- |
| **Cases/** | **Genotype** | | |  | **OR (95% CI)** |  | **p-value** |  | **OR (95% CI)** |  | **p-value** |
| **controls** | **SNP34091 n (%)** | | |  | **SNP34091** |  |  |  | **SNP34091** |  |  |
|  | **AA** | **AC** | **CC** |  | **CC vs. AA+AC** |  |  |  | **CC+AC vs. AA** |  |  |
| **Controls** | 243 (58.4) | 139 (33.4) | 34 (8.2) |  | 1.00 |  | - |  | 1.00 |  | - |
| **Women** | 120 (57.7) | 65 (31.3) | 23 (11.0) |  | 1.00 |  | - |  | 1.00 |  | - |
|  |  |  |  |  |  |  |  |  |  |  |  |
| **Breast cancer^a^** | 141 (64.4) | 66 (30.1) | 12 (5.5) |  | 0.40 (0.19-0.85) |  | 0.018 |  | 0.72 (0.48-1.08) |  | 0.116 |

^a^ Calculations with female controls only, age adjusted
